# Impact of Stress Echocardiography on Aortic Valve Stenosis Management

**DOI:** 10.3390/jcm13123495

**Published:** 2024-06-14

**Authors:** Andreas Synetos, Konstantina Vlasopoulou, Maria Drakopoulou, Anastasios Apostolos, Nikolaos Ktenopoulos, Odysseas Katsaros, Theofanis Korovesis, George Latsios, Kostas Tsioufis

**Affiliations:** 1 First Department of Cardiology, National and Kapodistrian University of Athens, Hippokration General Hospital of Athens, 11527 Athens, Greece; mdrakopoulou@hotmail.com (M.D.); anastasisapostolos@gmail.com (A.A.); nikosktenop@gmail.com (N.K.); odykatsaros@gmail.com (O.K.); faniskorovesis@gmail.com (T.K.);; 2 School of Medicine, European University of Cyprus, 2404 Egkomi, Cyprus

**Keywords:** aortic valve stenosis, stress echocardiography, stress test, asymptomatic severe aortic stenosis, low-flow, low-gradient aortic stenosis, prosthetic aortic valve, valvular heart disease

## Abstract

Rest and stress echocardiography (SE) play a fundamental role in the evaluation of aortic valve stenosis (AS). According to the current guidelines for the echocardiographic evaluation of patients with aortic stenosis, four broad categories can be defined: high-gradient AS (mean gradient ≥ 40 mmHg, peak velocity ≥ 4 m/s, aortic valve area (AVA) ≤ 1 cm^2^ or indexed AVA ≤ 0.6 cm^2^/m^2^); low-flow, low-gradient AS with reduced ejection fraction (mean gradient < 40 mmHg, AVA ≤ 1 cm^2^, left ventricle ejection fraction (LVEF) < 50%, stroke volume index (Svi) ≤ 35 mL/m^2^); low-flow, low-gradient AS with preserved ejection fraction (mean gradient < 40 mmHg, AVA ≤ 1 cm^2^, LVEF ≥ 50%, SVi ≤ 35 mL/m^2^); and normal-flow, low-gradient AS with preserved ejection fraction (mean gradient < 40 mmHg, AVA ≤ 1 cm^2^, indexed AVA ≤ 0.6 cm^2^/m^2^, LVEF ≥ 50%, SVi > 35 mL/m^2^). Aortic valve replacement (AVR) is indicated with the onset of symptoms development or LVEF reduction. However, there is often mismatch between resting transthoracic echocardiography findings and patient’s symptoms. In these discordant cases, SE and CT calcium scoring are among the indicated methods to guide the management decision making. Additionally, due to the increasing evidence that in asymptomatic severe aortic stenosis an early AVR instead of conservative treatment is associated with better outcomes, SE can help identify those that would benefit from an early AVR by revealing markers of poor prognosis. Low-flow, low-gradient AS represents a challenge both in diagnosis and in therapeutic management. Low-dose dobutamine SE is the recommended method to distinguish true-severe from pseudo-severe stenosis and assess the existence of flow (contractile) reserve to appropriately guide the need for intervention in these patients.

## 1. Introduction

Aortic stenosis (AS) is the most common primary valve lesion requiring surgery or transcatheter intervention in Europe and North America, with a rapidly rising prevalence due to the ageing population [1]. Transthoracic echocardiography (TTE) plays a key role in the initial evaluation of patients with known or suspected aortic stenosis and the confirmation of the diagnosis and its severity. The echocardiographic evaluation of patients with aortic stenosis depends on measurement of mean pressure gradient, peak transvalvular velocity (Vmax), and valve area (AVA) [1,2]. Valve area is theoretically the ideal index for assessing severity, but there are several technical limitations. Therefore, additional parameters such as functional status, stroke volume, Doppler velocity index, degree of valve calcification, left ventricular (LV) function, LV hypertrophy, flow conditions, and the adequacy of arterial blood pressure (BP) control should be taken into consideration for clinical decision making [2].

According to the current guidelines for the echocardiographic evaluation of patients with aortic stenosis, four broad categories can be defined (Table 1): high-gradient aortic stenosis (mean gradient ≥ 40 mmHg, peak velocity ≥ 4 m/s, aortic valve area (AVA) ≤ 1 cm^2^ or indexed AVA ≤ 0.6 cm^2^/m^2^); low-flow, low-gradient aortic stenosis with reduced ejection fraction (mean gradient < 40 mmHg, AVA ≤ 1 cm^2^, left ventricle ejection fraction (LVEF) < 50%, stroke volume index (SVi) ≤ 35 mL/m^2^); low-flow, low-gradient aortic stenosis with preserved ejection fraction (mean gradient < 40 mmHg, AVA ≤ 1 cm^2^, LVEF ≥ 50%, SVi ≤ 35 mL/m^2^); and normal-flow, low-gradient aortic stenosis with preserved ejection fraction (mean gradient < 40 mmHg, AVA ≤ 1 cm^2^, indexed AVA ≤ 0.6 cm^2^/m^2^, LVEF ≥ 50%, SVi > 35 mL/m^2^), which is usually equivalent to only moderate aortic stenosis [1,2].

The timing of intervention is determined by the development of symptoms or reduction in LVEF. However, there are cases in which the severity of the stenosis is uncertain, for example in the case of low-gradient severe AS based on AVA, or there is a mismatch between TTE findings and clinical presentation. In those cases, stress echocardiography (SE) can provide important complementary information helping clinical decision making [1,2]. In patients with severe aortic stenosis presenting as asymptomatic, SE may unmask symptoms and reduced exercise tolerance, whereas in symptomatic patients with non-severe stenosis or inconclusive TTE findings, SE may regrade AS severity, redefining the need for intervention and giving additional prognostic information [1,2,3,4].

The aim of this review is to summarise the existing evidence for the use of stress echocardiography in the diagnostic algorithm of severe aortic valve stenosis and its impact on the management decision making.

## 2. Stress Echocardiography Method

Valve stress echocardiography is performed with either exercise or dobutamine, depending on the clinical indication [5]. Exercise SE using treadmill or supine bicycle is the stressor of choice in asymptomatic severe aortic stenosis [1,2,3,4,5]. Exercise is physiological; it preserves the integrity of the electromechanical response and provides valuable information regarding functional status, helping assessing symptoms and exercise tolerance. Performing echocardiography during exercise also allows associating symptoms with cardiovascular workload; wall motion abnormalities; and haemodynamic responses, such as pulmonary pressure and changes in transvalvular flow and gradient. Treadmill exercise has the major disadvantage of allowing only pre- and post-stress image acquisition, while changes in recorded parameters are transient, immediately decreasing with cessation of exercise and lasting shortly, so the consequences of exercise can be underestimated or even missed. Supine bicycle exercise allows image acquisition at any stage during the SE and is technically easier than treadmill exercise [4,5]. The commonly used treadmill protocols are the Bruce and modified Bruce protocols [4]. When supine bicycle is used, the protocol begins with an initially low workload (0, 25, or 50 W depending on patient’s age, expected exercise tolerance, and pathology) and then increases in steps, usually by 25 W every 2–3 min [4,5]. For the assessment of exercise tolerance or contractile reserve, a modification with more gradual increase in workload can be used. The predicted maximum workload is 2.5 W/kg in women and 3 W/kg in men between 21 and 30 years old, minus 10% for each added decade. The workload achieved depends not only on the valve disease severity but also on the patient’s physical status and familiarity with the test [5].

Dobutamine SE is currently the recommended method for assessment of low-flow, low-gradient AS [1,2,3,4,5]. This is because of concerns that in asymptomatic, not physically active patients, symptoms or a low exercise tolerance can limit the exercise-induced contractile recruitment, preventing the correct assessment of both stenosis severity and flow reserve [5]. For AS severity grading, a low-dose dobutamine infusion protocol is used, starting from 5, up to 20 μg/kg/min titrated upward in steps of 2.5–5 μg/kg/min every 5–8 min. The test is terminated when the target increase in flow (20% increase in left ventricular outflow tract (LVOT)—derived stroke volume) is achieved. After each dobutamine dose increase, a period of 2–3 min is allowed to ensure stabilisation of the haemodynamic status before starting the image acquisition [4].

During exercise, SE images should be acquired at least at baseline (rest), low workload (intermediate stage), and peak stress (Table 2) [5]. When treadmill exercise is used, post-exercise imaging is of great significance, and thus it is crucial to complete post-exercise imaging as soon as possible since wall motion changes, valve gradients, and pulmonary haemodynamics normalise quickly during recovery [4]. During dobutamine SE, images should be acquired at the end of every stage. Continuous live imaging is preferable if transient changes are possible. For example, in the case of low-flow AS, an initial rise in LVOT and transvalvular velocities due to contractile recruitment can be transient followed by a reduction in systolic function caused by ischemia, with a consequent drop in velocity [5].

For data acquisition, the comprehensive ABCDE SE protocol has been proven to be feasible and useful in patients with chronic coronary syndromes, allowing phenotype identification and risk-stratification considering their many vulnerabilities beyond coronary artery stenosis. The protocol consists of five steps: step A: regional wall motion abnormality detection for myocardial ischemia, step B: B-lines detection by lung ultrasound for pulmonary congestion, step C: left ventricular contractile reserve by volumetric two-dimensional echocardiography, step D: coronary flow velocity reserve in mid-distal left anterior descending coronary artery with pulsed-wave Doppler estimating coronary microvascular reserve, and step E: assessment of sympathetic cardiac autonomic reserve with a one-lead electrocardiogram [6]. In valvular heart disease, it has been proposed that ABCDE SE may be integrated and enriched to assess transvalvular gradients (step G). This step includes evaluation of the Vmax, trans-aortic maximal, and mean gradients using continuous-wave Doppler techniques, evaluation of integral flow in the LVOT using pulsed-wave Doppler methods, and calculation of the stroke volume and AVA using the continuity equation method [7]. Each step is important in forming the outcome and represents a potential therapeutic target, inducing personalised risk stratification and tailored therapy approaches [6]. The reason for stopping the test, total exercise time, and maximum workload, as well as non-imaging parameters such as blood pressure, heart rate, arrythmias, and patient’s symptoms (angina, breathlessness, pre-syncope, or syncope), must also be reported [4,5]. The protocol is further tailored to each individual patient accordingly to the indication of the test.

Clinical conditions such as atrial fibrillation or other significant arrhythmias, uncontrolled hypertension, severe left ventricular hypertrophy, significant mitral regurgitation, significant coronary artery disease with inducible ischemia, or poor acoustic window due to obesity or chronic obstructive pulmonary disease represent potential limitations of the accuracy and/or utility of SE in AS assessment [1,5,8].

## 3. Asymptomatic Severe Aortic Stenosis

Symptomatic severe aortic stenosis has dismal prognosis, and early intervention is strongly recommended [2]. The most common initial symptoms are exertional dyspnoea and decreased exercise tolerance. The early recognition of these symptoms followed by treatment of the valve stenosis is of high significance so that more severe symptoms like HF, syncope, and angina can be avoided [1]. However, some patients with severe AS may not report any symptoms at rest as they have adapted their activity to symptoms development [3]. In these cases, exercise testing with appropriate physician supervision and close ECG and blood pressure monitoring is safe [4] and may reveal a concealed symptomatic severe AS [1,2,3], providing additionally prognostic information beyond the need for intervention [4]. According to the current guidelines, in patients with severe aortic stenosis, the onset of symptoms and/or impaired LV function with LVEF < 50% (class I in American College of Cardiology (ACC)/American Heart Association (AHA) 2020 and European Society of Cardiology (ESC)/ European Association for Cardio-Thoracic Surgery (EACTS) 2021) or LVEF < 55% (class IIa in ESC/EACTS 2021) of no other cause represents a clear indication for aortic valve replacement (AVR) [1,2]. AVR is also recommended for those who are asymptomatic during normal activities but develop symptoms during exercise testing (class I in both guidelines) [1,2].

In symptomatic patients with severe AS, exercise testing is contraindicated because of the risk of severe haemodynamic compromise [1], whereas in asymptomatic patients with severe AS, exercise testing is indicated to assess physiological changes with exercise and to confirm the absence of symptoms. For the assessment of asymptomatic severe AS, exercise SE with treadmill or supine bicycle can be used. The minimum acquired measurements include Vmax, transaortic mean pressure gradient, LVEF assessment by biplane Simpson from apical four- and two-chamber views, and pulmonary artery systolic pressure (PASP) estimation. The continuous wave Doppler should ideally be performed from the window from which the maximum velocity was obtained at rest [4]. Non-imaging parameters including blood pressure and patient’s symptoms should also be recorded [4,5]. Parameters that characterise an abnormal exercise response are ventricular arrhythmia; systolic blood pressure drop or failure to rise; ST-segment depression; induced regional wall motion abnormalities; reduced contractile reserve; impaired rest or exertion LV longitudinal function; exercise-induced pulmonary hypertension (PASP > 60 mmHg); and development of symptoms like angina, breathlessness, pre-syncope, or syncope [5].

Markers of poor prognosis in exercise SE include an increase in mean gradient by ≥18–20 mmHg [9], the absence or limitation of LV contractile reserve (decrease or no increase in LVEF suggesting subclinical LV dysfunction), and exercise-induced pulmonary hypertension. The increase in mean gradient during exercise can be either because of the presence of more severe AS or a non-compliant rigid aortic valve [4]. The increase in mean gradient is the only one of the three prognostic markers considered as a predictor of symptom development and adverse outcomes and may be an indication for early intervention in asymptomatic patients if procedural risk is low [2]. The lack of LV contractile reserve with exercise may reflect more advanced disease with LV afterload mismatch and/or exercise-induced exhaustion of coronary flow reserve. For the detection of subclinical LV systolic dysfunction, LVEF has low sensitivity. On the other hand, assessment of LV global longitudinal strain (LVGLS) has shown to be more accurate in predicting the occurrence of symptoms, exercise intolerance, and cardiac events in asymptomatic patients with severe AS and preserved LVEF [4]. LVGLS < 14.7% (<15% according to ESC/EACTS 2021) may help identifying patients with severe asymptomatic aortic stenosis who are at higher risk of clinical deterioration or premature mortality [2,10]. In a recent study, in patients with severe AS undergoing transcatheter aortic valve implantation (TAVI), right ventricular (RV) GLS but not LVGLS was reduced in non-survivors compared to survivors, differentiated non-survivors from survivors, was independently associated with mortality, and exhibited potential incremental value for outcome prediction, making RVGLS more suitable than LVGLS for risk stratification in AS and timely valve replacement [11].

Concerning non-imaging parameters, besides symptom occurrence, asymptomatic patients with severe AS who present a fall in systolic blood pressure ≥10 mmHg from baseline to peak exercise, or a significant decrease in exercise tolerance as compared with age and sex normal standards, seem to have high rate of symptom onset within 1 to 2 years (about 60–80%) [1]. These patients have a class IIa recommendation for AVR according to ACC/AHA 2020 guidelines [1], whereas a class IIa recommendation for AVR in ESC/EACTS 2021 guidelines is suggested for patients who present a fall in systolic blood pressure of >20 mmHg [2]. Management of patients with a lack of appropriate rise in BP with exercise is less clear.

In the absence of adverse prognostic features in asymptomatic patients with severe AS, watchful waiting has generally been recommended until symptoms onset [2]. The follow-up should be at least every 6 months to allow earliest symptom detection and any change in echocardiographic parameters, particularly LVEF. If symptoms are doubtful during follow-up, exercise testing should be used [2]. For patients with severe AS who develop pulmonary hypertension or have limited contractile reserve, the clinical and echocardiographic follow-up should be closer. The same should happen for patients with moderate AS having a marked increase in pressure gradient during exercise [4,9] or multiple cardiovascular risk factors [12]. Additionally, it has been shown that in patients with non-severe AS, an increase in E/e’ ratio of ≥15% during exercise is related to a very high risk for adverse cardiac events in the medium and short-term and they should potentially have a follow-up every 3–6 months [12].

An abnormal exercise SE in clinically asymptomatic patients is associated with higher risk of major adverse cardiac events and sudden death. These risks may be higher than the risk for surgical intervention, favouring AVR [13]. There is increasing evidence that in asymptomatic severe aortic stenosis, an early AVR instead of conservative treatment is associated with better outcomes [14,15,16,17,18] by reducing all-cause, cardiovascular, and non-cardiovascular mortality [16,17] without increasing any procedure-related clinical outcomes [16]. The AVATAR trial showed that in asymptomatic patients with severe AS, early surgery reduced a primary composite of all-cause mortality, acute myocardial infarction, stroke, or unplanned hospitalisation for heart failure compared with conservative treatment, providing support for early SAVR once AS becomes severe, regardless of symptoms [19]. The RECOVERY trial showed that in patients with very severe aortic stenosis, there was significant reduction in operative mortality or death from cardiovascular causes among those who underwent early SAVR compared with conservative management [20]. Furthermore, an analysis of the patients from the PARTNER trials showed that the extent of cardiac damage before AVR has an important impact on patient’s health status even after AVR, which means that the development of strategies to minimise the cardiac damage before AVR is needed [21]. This could happen with early intervention at the time AS becomes severe before extravalvular cardiac damage initiates. However, early AVR is not yet recommended from the guidelines. Further randomised trials (EARLY TAVR (NCT03042104), DANAVR [NCT03972644], EASY-AS (NCT04204915), EVOLVED (NCT03094143)) will help determine future recommendations, probably in favour of early AVR in selected populations.

## 4. Low-Flow, Low-Gradient Aortic Stenosis

Among patients screened for AS severity using TTE, up to 40% present discordant grading, having a small AVA along with low gradient [22]. Low-flow, low-gradient (LFLG) AS may occur with reduced (classical LFLG AS) or preserved LVEF (paradoxical LFLG AS). In both cases, the reason of the decrease in gradient is a reduction in transvalvular flow [4]. In symptomatic patients, revaluation of valve disease severity based on flow-dependent changes or on its dynamic component is required [5]. SE is indicated in LFLG AS to confirm AS severity and eventual indication for AVR [1,2,3,4,5].

### 4.1. Classical Low-Flow, Low-Gradient Aortic Stenosis

In LFLG AS with reduced LVEF (<50%), LV systolic dysfunction can be caused by either afterload mismatch due to truly severe AS or by primary myocardial dysfunction with only moderate AS [1]. The use of low-dose dobutamine SE or the evaluation of aortic valve calcium score by cardiac computed tomography (CT) is recommended to distinguish between true and pseudo-severe aortic stenosis [1,2,4,5,23]. Moreover, SE can also be utilised to assess the existence of flow (contractile) reserve and to detect inducible ischemia, as in calcific AS, coexistence of coronary artery disease is common [1,2,4,5].

**Scheme 1:**
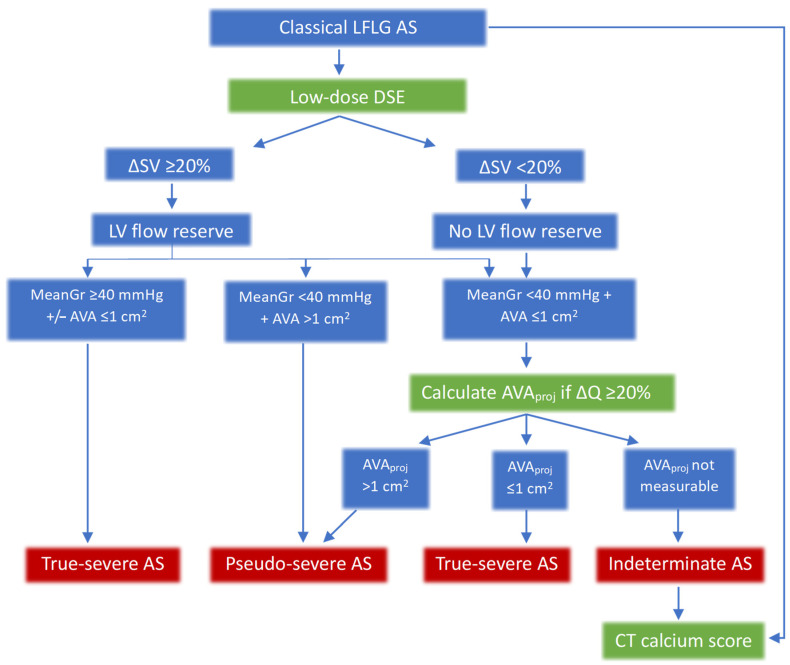
Classical low-flow, low-gradient aortic stenosis categorisation according to dobutamine stress echocardiography findings. LFLG, low-flow low-gradient; AS, aortic stenosis; DSE, dobutamine stress echocardiography; ΔSV, stroke volume increase; LV, left ventricle; MeanGr, mean pressure gradient; AVA, aortic valve area; ΔQ, change in flow rate, AVAproj, projected AVA; CT, computed tomography.

During dobutamine SE, Doppler tracings and LV images are obtained at rest and at each dobutamine infusion stage. The minimum acquired dataset includes aortic valve CW Doppler, LVOT PW Doppler (placing the sample volume at the same position in the LVOT as much as possible at every stage is optimal), views of the LV in parasternal long-axis, and apical two- and four-chamber. LV function with changes in EF or global strain, flow reserve (increase in stroke volume ≥20%), changes in pressure gradients, and AVA should be also assessed. Three response patterns are possible (Scheme 1). To define them, the first step is to determine the presence of flow reserve, defined as a relative increase in stroke volume (SV) >20%. If there is flow reserve, AVA remains <1 cm^2^, and the mean gradient exceeds 40 mmHg, the stenosis is considered true severe. If there is flow reserve but AVA increases >1 cm^2^ while the transaortic gradient remains low, the stenosis is pseudo-severe. If there is no flow reserve, there is still uncertainty about the stenosis severity.

The low sensitivity of the dobutamine SE criteria due to persistence of a low-flow state during stress and persistent discordant grading of AS severity using mean gradient and AVA represent its most important limitation [23]. The lack of stroke volume increase during dobutamine SE can result from afterload mismatch due to an imbalance between the severity of the stenosis and myocardial reserve, inadequate increase in myocardial blood flow due to associated coronary artery disease, and/or irreversible myocardial damage due to previous myocardial infarction or extensive myocardial fibrosis [4]. The TOPAS study showed that, in these cases, the application of a lower cutoff value for peak stress mean gradient 35 mmHg can improve the sensitivity of dobutamine SE for the identification of true-severe AS [23]. Moreover, as AVA measured at low flow rate is not a good prognostic marker and therefore not a good diagnostic marker for truly severe AS [24,25], calculation of the projected AVA at normal flow rate (Q) (i.e., 250 mL/s) should be considered to correctly classify AS severity and to guide patient management [4,23,25,26]. The calculation formula is

$$Projected\;AVA=AVArest+\left(\frac{\Delta AVA}{\Delta Q}\right)\times \left(250-Qrest\right),$$

where AVArest and Qrest are the AVA and mean transvalvular flow rate measured at rest, and ΔAVA and ΔQ are the absolute changes in AVA and Q measured during dobutamine SE. If the projected AVA is ≤1 cm^2^, the stenosis is severe [4,23,26]. Projected AVA indexed to body surface can also be used with a value ≤0.6 cm^2^/m^2^, suggesting severe AS [23]. However, if the ΔQ is <20%, the estimation of the projected AVA may not be reliable [4,23,26]. In case projected AVA is not measurable, the evaluation of aortic valve calcium score by cardiac CT is indicated to corroborate AS severity [1,2,4,23]. The aortic stenosis is highly likely for calcium score for >3000 Agatston units in men and >1600 in women, likely for >2000 in men and >1200 in women, and unlikely for <1600 in men and <800 in women [2].

For symptomatic patients with severe LFLG AS with reduced LVEF (<50%) and evidence of flow reserve, there is a class I recommendation for AVR in both ACC/AHA 2020 and ESC/EACTS 2021 guidelines [1,2]. For symptomatic patients with severe LFLG AS with reduced LVEF (<50%) without flow reserve, the recommendation from ACC/AHA 2020 guidelines for AVR remains class I [1]; however, in ESC/EACTS 2021 guidelines, AVR in these patients has a weaker, class IIa recommendation [2]. For patients with pseudo-severe AS, optimal medical treatment and close follow-up with rest TTE are recommended [1,2,4].

It has been shown that the presence or absence of flow reserve does not influence prognosis of patients undergoing TAVI or surgical aortic valve replacement (SAVR) [2,27]. Although patients without flow reserve show increased procedural mortality [2,28] (thus own a weaker recommendation for AVR), both modes of intervention have been shown to improve ejection fraction and clinical outcomes [2,27]. Thus, the absence of flow reserve should not preclude consideration for SAVR or TAVI [4]. The results of the TOPAS-TAVI Registry analysis showed that TAVI was associated with good periprocedural outcomes in patients with LFLG AS and the absence of flow reserve at dobutamine SE was not associated with any negative effect on clinical outcomes or LVEF changes at follow-up [29,30]. Also, according to a meta-analysis of five observational studies, the absence of flow reserve in patients with LFLG AS and reduced LVEF who underwent TAVI did not appear to have an impact on all-cause mortality [31]. These, in addition to the increased burden of co-morbidities of these patients, make TAVI an attractive option in this population as a less invasive, better tolerated procedure, even for those with severe left ventricular dysfunction [4,29,30,31,32].

AS patients with LFLG AS and reduced LVEF often have a poor prognosis with medical treatment but increased operative mortality with AVR; both guidelines suggest individualised decisions for their management [1,2]. Keeping in mind that the presence or absence of flow reserve does not represent an independent predictor of clinical outcomes and that the SE demonstrated lack of flow reserve does not predict lack of LVEF recovery, additional parameters for risk stratification are needed. The transaortic flow rate (i.e., stroke volume/LV ejection time) is, besides the AVA, the main physiological determinant of the increase in transvalvular pressure gradient and has shown to predict mortality better than stroke volume in low-gradient aortic stenosis [33,34]. A multicentre study showed that stress transaortic flow rate during low-dose dobutamine stress echocardiography was useful for the detection of both AS severity and flow reserve and was associated with improved prediction of outcome following AVR. Patients with severe aortic valve stenosis based on aortic valve area <1 cm^2^ at stress transaortic flow rate ≥210 mL/s showed improved outcome with aortic valve intervention compared with medical therapy [35]. According to results from the TOPAS study, in patients with LFLG AS, using two-dimensional strain parameters measured by speckle tracking at rest and during dobutamine SE to assess the extent of myocardial impairment are strong predictors of outcome. Left ventricular global longitudinal strain (LV GLS) has shown to be superior to other parameters of LV function for risk stratification. LV GLS is severely impaired in these patients at rest but may improve with dobutamine stress. Low rest GLS is a strong risk factor for mortality in patients treated either conservatively or by AVR, whereas stress GLS seems to be superior to rest GLS to predict outcomes in these patients [36,37]. Additionally, the reduced RV free wall longitudinal strain (RVLS) seems to be independently associated with increased risk of mortality, with stress RVLS providing incremental prognostic value beyond that obtained from rest RVLS [38]. All these parameters can assist risk stratification and individualised decisions concerning the management of patients with classical LFLG AS, complementary to their comorbidities, the degree of valve calcification, the extent of coronary artery disease (CAD), and the feasibility of revascularisation.

### 4.2. Paradoxical Low-Flow, Low-Gradient Aortic Stenosis

LFLG AS with preserved LVEF typically concerns hypertensive elderly patients with small LV size and marked hypertrophy. Low flow can also result from conditions associated with low stroke volume like moderate or severe mitral regurgitation, severe tricuspid regurgitation, severe mitral stenosis, and large ventricular septal defect with severe RV dysfunction. For the diagnosis of severe aortic stenosis in these patients, careful exclusion of measurement errors and other explanations for the echocardiographic findings are required [2]. Doppler velocity data should ideally be recorded when the patient is normotensive, as the assessment of AS severity when the patient is hypertensive may underestimate or, less often, overestimate the stenosis severity. Systemic hypertension imposes a second pressure load on the LV on top of valve obstruction, resulting in a lower forward stroke volume and lower transaortic pressure gradient [1]. LV hypertrophy in the absence of coexistent hypertension, reduced LV GLS of no other cause, and the presence or absence of typical symptoms of no other explanation should also been taken into consideration [1,2].

**Scheme 2:**
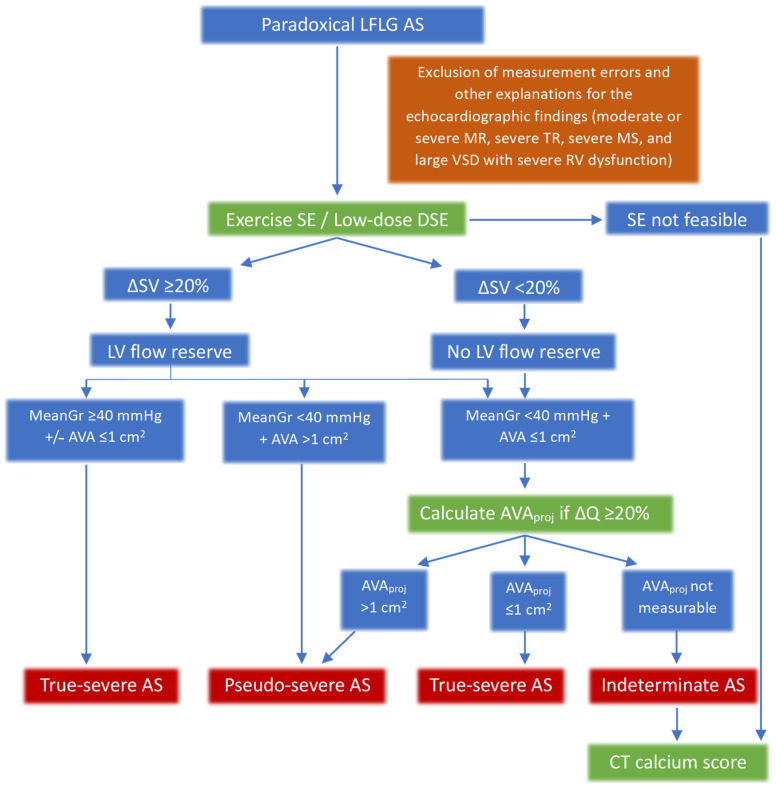
Paradoxical low-flow, low-gradient aortic stenosis categorisation according to dobutamine stress echocardiography findings. LFLG, low-flow low-gradient; AS, aortic stenosis; MR, mitral regurgitation; TR, tricuspid regurgitation; MS, mitral stenosis; VSD, ventricular septal defect; RV, right ventricle; SE, stress echocardiography; DSE, dobutamine stress echocardiography; ΔSV, stroke volume increase; LV, left ventricle; MeanGr, mean pressure gradient; AVA, aortic valve area; ΔQ, change in flow rate, AVAproj, projected AVA; CT, computed tomography.

Similar to classical LFLG AS, about one-third of patients with paradoxical LFLG AS have pseudo-severe stenosis due to incomplete opening of a moderately stenotic valve. If rest echocardiographic measurements, having taken into account all of the above, still suggest a severe LFLG AS with preserved LVEF, then as in classical LFLG AS, true severe AS should be distinguished from pseudo-severe stenosis. Exercise (in patients without or with mild or unclear symptoms) or low-dose dobutamine (in symptomatic patients) SE may be useful in patients with paradoxical LFLG AS to verify stenosis severity, using the same parameters and criteria as those for classical LFLG AS, including projected AVA [4,39] (Scheme 2). Yet, in some cases, due to the presence of LV restrictive physiology pattern, dobutamine SE can be inconclusive or not feasible in these patients. The guidelines recommend cardiac CT for the assessment of the degree of valve calcification using the same thresholds as in inconclusive cases with LFLG AS and reduced LVEF [1,2,4]. Valve calcium scoring predicts disease progression and clinical events and may be useful in assessing the severity of aortic stenosis in patients with low valve gradient in combination with the geometric assessment of valve area [2].

In symptomatic patients with paradoxical LFLG AS in whom there has been careful confirmation that the aortic stenosis is severe and the AS is the most likely cause of their symptoms, AVR has a class IIa recommendation in ESC/EACTS 2021 guidelines [2], whereas in ACC/AHA 2020 guidelines, AVR has a class I recommendation [1]. The higher class of recommendation in ACC/AHA 2020 guidelines is because AVR appears beneficial, with an increase in stroke volume and improved survival as compared with medical therapy in these patients [1]. Also, although the survival rate after TAVI is generally lower in patients with LFLG AS than in patients with classic high-gradient AS, among patients with LFLG, TAVI outcomes have shown to be similar—either they have reduced or preserved LVEF [40].

## 5. Conclusions

**Scheme 3:**
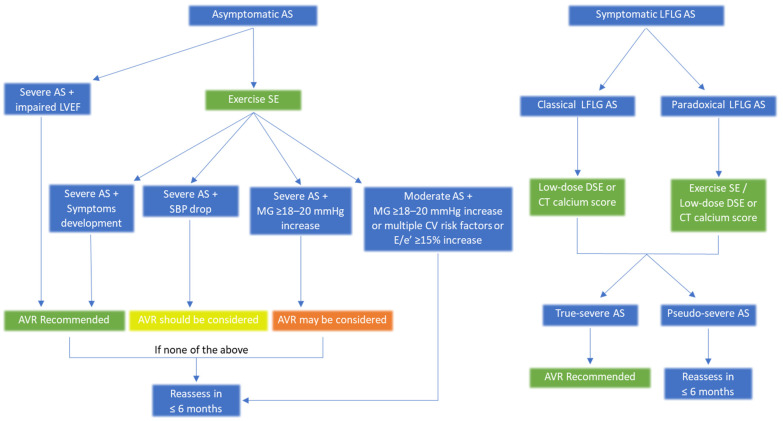
Role of stress echocardiography in aortic valve stenosis management. AS, aortic stenosis; LFLG, low-flow low-gradient; LVEF, left ventricle ejection fraction; SE, stress echocardiography; DSE, dobutamine stress echocardiography; SBP, systolic blood pressure; MG, mean pressure gradient; VC, cardiovascular; CT, computed tomography; AVR, aortic valve replacement.

The indications of SE in non-ischaemic heart disease evolve continuously. Given the heterogeneity of the aortic valve stenosis population, SE plays a crucial role in the individualised management of these patients. In discordant cases, SE may regrade AS severity, unmask symptoms, drive the follow-up, give additional prognostic information, and finally redefine the need for intervention (Scheme 3). For patients with severe AS who are symptomatic and/or have impaired LV function of no other cause, AVR is recommended. For patients with severe AS who are asymptomatic during normal activities, SE is indicated to define the need for intervention. If symptoms develop during exercise testing, AVR is recommended. If a fall in systolic blood pressure ≥10 mmHg (ACC/AHA guidelines 2020) or >20 mmHg (ESC/EACTS guidelines 2021) from baseline to peak exercise presents, AVR should be considered [1,2]. Additionally, if an increase in mean gradient by ≥18–20 mmHg presents, AVR may be considered if procedural risk is low. In the absence of the above, watchful waiting until symptoms onset is recommended. The follow-up should take place at least every 6 months, including exercise testing if symptoms are doubtful, or closer if patients develop pulmonary hypertension or have limited contractile reserve. Patients with non-severe AS who present marked increase (≥18–20 mmHg) in pressure gradient during exercise or have multiple cardiovascular risk factors should also be follow-up closely, at least every 6 months. For symptomatic patients with LFLG AS, dobutamine SE or CT calcium score is recommended to confirm the severity of the disease and define the need for intervention. If SE proves that the stenosis is true severe, AVR is recommended. In case of pseudo-severe AS, patients should receive optimal medical treatment and have a close follow-up. Further evidence from larger-scale studies is needed for expanding SE indications according to its impact on patient outcomes.

## Figures and Tables

**Table 1:** Aortic stenosis categorisation according to echocardiographic findings.

Aortic Stenosis Category	Echocardiographic Parameters				
	Mean Gradient (mmHg)	Vmax (m/s)	AVA (cm^2^), [AVAi (cm^2^/m^2^)]	LVEF (%)	SVi (mL/m^2^)
High-gradient AS	≥40	≥4	≤1 [≤0.6]	- *	- *
LFLG AS with reduced LVEF (classical LFLG AS)	<40	<4	≤1	<50	≤35
LFLG AS with preserved LVEF (paradoxical LFLG AS)	<40	<4	≤1	≥50	≤35
NFLG AS with preserved LVEF	<40	<4	≤1 [≤0.6]	≥50	>35

* Severe high-gradient aortic stenosis can be assumed irrespective of LV function and flow conditions. Vmax, peak velocity; AVA, aortic valve area; AVAi, aortic valve area indexed; LVEF, left ventricular ejection fraction; SVi, stroke volume index; AS, aortic stenosis; LFLG, low-flow low-gradient; NFLG, normal-flow low-gradient.

**Table 2:** Stress echocardiography protocol in aortic valve stenosis.

Data Acquisition		2D		STE	Lung US	CW Doppler	PW Doppler		CF Doppler	ECG	BP	Symptoms
Parameters		RWMA	EDV, ESV, LVEF	GLS	B-Lines	Vmax, Aortic MG, Aortic PG, SPAP	LVOT Flow	LAD CFR	MR	HR		
Stages	Baseline	+	+	+	+	+	+	+	+	+	+	+
	Low workload	+	+			+	+			+	+	+
	Peak exercise	+	+	+		+	+	+	+	+	+	+
	Recovery	+	+		+					+	+	+

+, included; 2D, two-dimensional echocardiography; STE, speckle tracking echocardiography; US, ultrasound; CW, continuous-wave; PW, pulsed-wave; CF, colour-flow; ECG, electrocardiogram; BP, blood pressure; RWMA, regional wall motion abnormalities; EDV, end-diastolic volume; ESV, end-systolic volume; LVEF, left ventricle ejection fraction; GLS; global longitudinal strain; Vmax, peak transaortic velocity; MG, mean pressure gradient; PG, peak pressure gradient; SPAP, systolic pulmonary artery pressure; LVOT, left ventricle outflow tract; LAD CFR, left ascending artery coronary flow reserve; MR, mitral regurgitation; HR, heart rate.
